# A Quality Improvement Project to Improve the Weekly Ward Review by the Consultant on the General Adult Inpatient Wards in Mersey Care NHS Foundation Trust – An Evaluation of Change

**DOI:** 10.1192/bjo.2025.10375

**Published:** 2025-06-20

**Authors:** Ankita Vinjamuri, Jonathan Tjong A Tjoe, Ranjan Baruah, Roweida Sammour, Declan Hyland

**Affiliations:** Mersey Care NHS Foundation Trust, Liverpool, United Kingdom

## Abstract

**Aims:** Ward reviews are a pivotal component of patient care in psychiatric inpatient settings, offering a structured opportunity for patients to engage with their treatment team and contribute to their treatment plan. Ward reviews are essential for discussing treatment progress, making necessary adjustments to care plans, and addressing patient concerns. The aim of this quality improvement project was to assess and improve the experience of ward reviews for inpatients on the Trust’s general adult inpatient wards by ensuring inpatients are well-informed about the timing of and attendees for their ward reviews and help them prepare effectively for their ward review.

**Methods:** Following the implementation of a “ward review sign” and ward review template on one of the Trusts’ general adult inpatient wards, the patients on the ward were surveyed to review whether the implementation of both interventions had improved patient awareness of when their next ward review was and patient satisfaction with their ward review.

**Results:** All 20 of the patients on the general adult inpatient ward completed the survey. Patient awareness of when their next ward review was increased from 50% pre-intervention to 70% post-intervention. Patient awareness of which staff would be present for the ward review increased from 25% pre-intervention to 65% post-intervention. Patient preparedness for the ward review increased from 40% pre-intervention to almost 50% post-intervention. Overall patient satisfaction with the ward review increased from 40% pre-intervention to 75% post-intervention.

**Conclusion:** Whilst it was clear that implementation of the two interventions had resulted in improvement in patient experience of the ward review, it was noted that the ward review sign was not updated on a weekly basis by the nursing team for every patient on the ward and that there was low utilisation of the ward review template for patients on the ward. This highlighted the challenge of implementing change and embedding this into the ward culture, particularly with nursing staff on the ward facing competing clinical tasks to be completed. The authors recommended a need for better communication between the medical and nursing staff on the ward. It was felt that proactive distribution of the ward proforma to the patient at the end of each patient’s ward review would help improve its utilisation. The nursing team was asked to allocate a dedicated member of staff to update the ward review sign in each patient’s bedroom on a once weekly basis. A need for re-evaluation of performance would then be required.

